# Long-term exposure to ambient air pollutants and increased risk of end-stage renal disease in patients with type 2 diabetes mellitus and chronic kidney disease: a retrospective cohort study in Beijing, China

**DOI:** 10.1007/s11356-023-31346-2

**Published:** 2023-12-20

**Authors:** Zhi Shang, Yue-Ming Gao, Zhen-Ling Deng, Yue Wang

**Affiliations:** 1 Department of Cardiology and Institute of Vascular Medicine, Peking University Third Hospital, Beijing, 100191 China; 2 State Key Laboratory of Vascular Homeostasis and Remodeling, Peking University, Beijing, China; 3 NHC Key Laboratory of Cardiovascular Molecular Biology and Regulatory Peptides, Peking University, Beijing, China; 4 Beijing Key Laboratory of Cardiovascular Receptors Research, Beijing, 100191 China; 5 Department of Nephrology, Peking University Third Hospital, 49 Huayuan North Road, Haidian District, Beijing, 100191 China

**Keywords:** Type 2 diabetes mellitus, Chronic kidney disease, End-stage renal disease, Air pollution, Long-term exposure, Cohort

## Abstract

Limited data have examined the association between air pollution and the risk of end-stage renal disease (ESRD) in patients with type 2 diabetes mellitus (T2DM) and chronic kidney disease (CKD). We aimed to investigate whether long-term exposure to air pollutants is related to the development of ESRD among patients with T2DM and CKD. A total of 1,738 patients with T2DM and CKD hospitalized in Peking University Third Hospital from January 1, 2013, to December 31, 2021 were enrolled in this study. The outcome was defined as the occurrence of ESRD. Data on six air pollutants (PM_2.5_, PM_10_, CO, NO_2_, SO_2,_ and O_3_) from 35 monitoring stations were obtained from the Beijing Municipal Ecological and Environmental Monitoring Center. Long-term exposure to air pollutants during the follow-up period was measured using the ordinary Kriging method. During a mean follow-up of 41 months, 98 patients developed ESRD. Multivariate logistic regression analysis showed that an increase of 10 μg/m^3^ in PM_2.5_ (odds ratio [OR] 1.19, 95% confidence interval [CI] 1.03–1.36) and PM_10_ (OR 1.15, 95% CI 1.02–1.30) concentration were positively associated with ESRD. An increase of 1 mg/m^3^ in CO (2.80, 1.05–7.48) and an increase of 1 μg/m^3^ in SO_2_ (1.06, 1.00–1.13) concentration were also positively associated with ESRD. Apart from O_3_ and NO_2_, all the above air pollutants have additional predictive value for ESRD in patients with T2DM and CKD. The results of Bayesian kernel machine regression and the weighted quantile sum regression all showed that PM_2.5_ was the most important air pollutant. Backward stepwise logistic regression showed that PM_2.5_ was the only pollutant remaining in the prediction model. In patients with T2DM and CKD, long-term exposure to ambient PM_2.5_, PM_10_, CO, and SO_2_ was positively associated with the development of ESRD.

**Supplementary Information:**

The online version contains supplementary material available at 10.1007/s11356-023-31346-2.

## Introduction

Diabetes mellitus (DM) has become a global health problem with many adverse outcomes (Zimmet et al. 2016). Globally, the estimated prevalence of DM in 2021 was 10.5% (536.6 million people), rising to 12.2% (783.2 million) in 2045 (Sun et al. 2022). China has become the country with the largest number of DM patients, of which more than 90% are type 2 diabetes mellitus (T2DM) (Wang et al. 2021). The increasing prevalence of DM aggravates the burden of chronic kidney diseases (CKD) and end-stage renal disease (ESRD) (GBD Chronic Kidney Disease Collaboration 2020). In developed countries, T2DM has become the leading cause of ESRD (Saran et al. 2020). In China, DM has become the second cause of ESRD after glomerulonephritis (Liu 2013).

ESRD is characterized by an irreversible decline in renal function and ultimately requires renal replacement therapy (RRT), including maintenance hemodialysis (HD), peritoneal dialysis (PD), and kidney transplantation, to maintain life. A population-based retrospective study involving over 25 million inhabitants showed that the incidence rate of chronic RRT among people with DM was almost six times higher than among people without DM (Claessen et al. 2021). T2DM-related ESRD not only leads to a reduction in life expectancy (Sattar et al. 2012) and quality of life (Chen et al. 2017) but also brings a heavy economic burden to patients and society (Chen et al. 2020).

Ambient air pollutants are a complex mixture of suspended particulate matters (PMs) and gases, such as PM with an aerodynamic diameter less than 2.5 μm (PM_2.5_) and less than 10 μm (PM_10_), nitrogen dioxide (NO_2_), carbon monoxide (CO), sulfur dioxide (SO_2_), and ozone (O_3_) (Shubham et al. 2022). Air pollution has been reported to be associated with chronic respiratory diseases (Annesi-Maesano et al. 2021), cardiovascular diseases (de Bont et al. 2022), stroke (Verhoeven et al. 2021), and cancers (Collatuzzo and Boffetta 2023), which is also a major contributor to global mortality (Cohen et al. 2017). In recent years, a growing body of evidence has shown that increased exposure to ambient air pollutants corresponds to an increased risk of CKD (Bowe et al. 2017, 2018; Lin et al. 2018, 2020a; Blum et al. 2020; Yang et al. 2017; Wang et al. 2020), renal function decline (Bowe et al. 2017, 2018; Wang et al. 2020; Chang et al. 2022), and ESRD (Bowe et al. 2017, 2018; Lin et al. 2020a, b). Air pollutants may cause kidney damage through various mechanisms, such as increasing blood pressure, aggravating oxidative stress and inflammatory responses, causing DNA damage, and inducing abnormal metabolic changes (Shubham et al. 2022).

Most of the evidence has been focused on PM_2.5_ (Bowe et al. 2018; Lin et al. 2018, 2020a, b; Blum et al. 2020) or PM_10_ (Bowe et al. 2017; Yang et al. 2017; Wang et al. 2020), and only a few studies investigate the effect of gaseous pollutants or multi-pollutants exposure on renal function (Bowe et al. 2017; Lin et al. 2018, 2020a; Chang et al. 2022). Furthermore, data regarding the association between multiple air pollutants and the risk of ESRD in patients with T2DM and CKD are lacking. Therefore, the current study aimed to investigate the association between long-term exposure to six types of air pollutants (PM_2.5_, PM_10_, CO, NO_2_, SO_2_, and O_3_) and the risk of ESRD in Chinese patients with T2DM and CKD.

## Materials and methods

### Study population

**Fig. 1:**
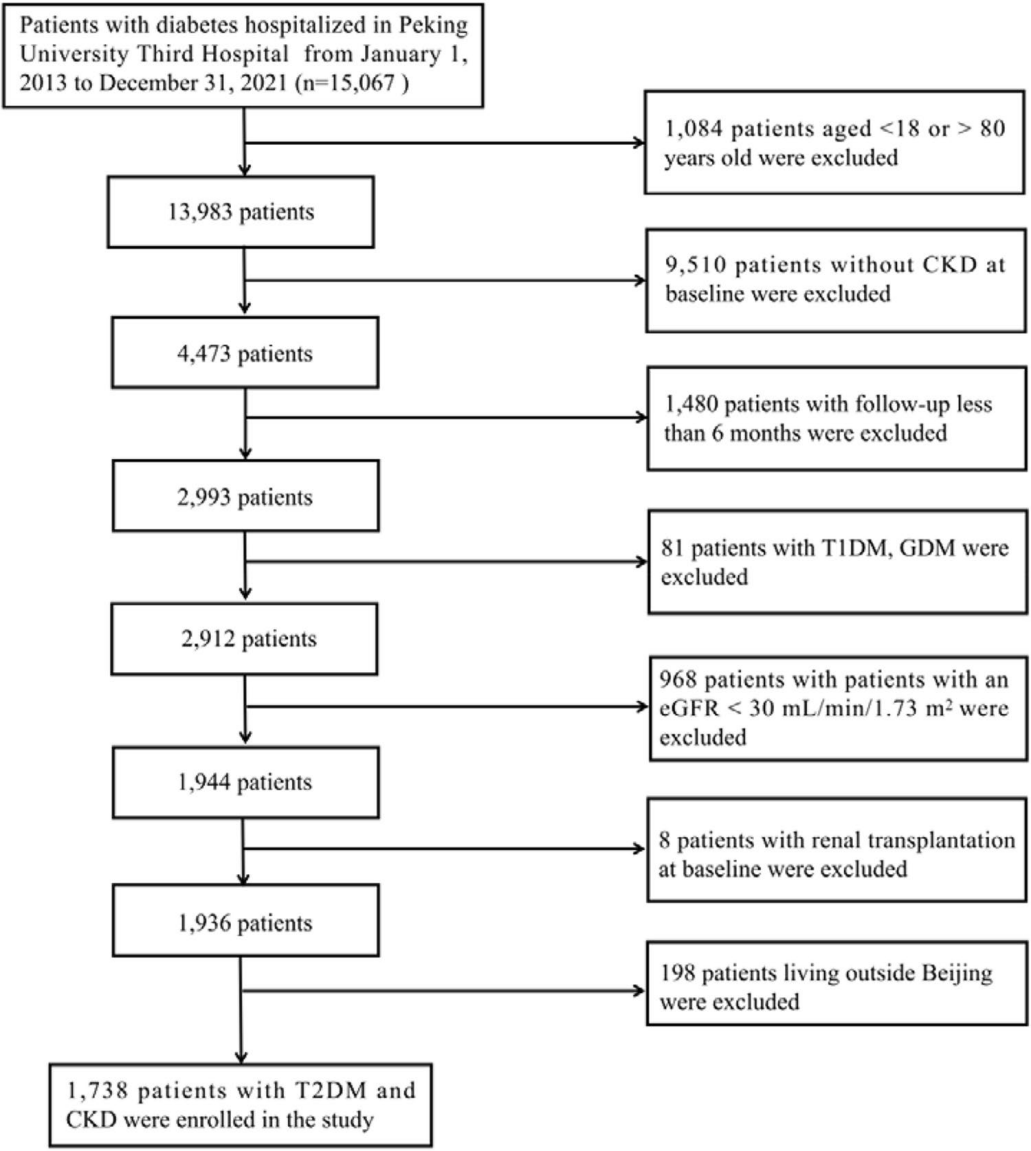
Enrollment flowchart for the study population

We enrolled 15,067 patients diagnosed with diabetes who were hospitalized at Peking University Third Hospital between January 2013 and December 2021 in Beijing, China. After excluding patients with age < 18 or > 80 years, without CKD at baseline, with a follow-up period of fewer than six months, with a diagnosis of type 1 diabetes mellitus (T1DM) or gestational diabetes mellitus (GDM), with an estimated glomerular filtration rate (eGFR) < 30 mL/min/1.73 m^2^, with a previous medical history of kidney transplantation, and with a permanent residence outside Beijing, a total of 1,738 patients with T2DM and CKD were finally included in this study. CKD was defined as a persistent decline in renal function (eGFR < 60 mL/min/1.73 m^2^) and/or the existence of albuminuria (urine protein ≥ 1 + in at least two out of three consecutive measurements within half a year) at baseline. The process for research population enrollment is described in Fig. 1.

### Health data and definitions

Clinical data were obtained from Peking University Third Hospital’s electronic medical records system. Patients’ information, such as basic clinical characteristics, medication history, main complications or comorbidities, important laboratory indicators, and a coordinated residential address, were included in this study. Basic clinical characteristics included age, age of DM onset, DM duration, sex, body mass index (BMI), blood pressure, and smoking status. Medication history included the usage of insulin, renin–angiotensin–aldosterone system (RAAS) inhibitors, or lipid-lowering drugs for treatment. Main complications or comorbidities included a medical history of hypertension, coronary artery disease (CAD), stroke, hyperlipidemia, heart failure (HF), or anemia. Laboratory indicators included hemoglobin (HGB), serum albumin (ALB), fasting blood glucose (FBG), glycated hemoglobin (HbA1c), total cholesterol (TC), triglyceride (TG), high-density lipoprotein cholesterol (HDL-C), low-density lipoprotein cholesterol (LDL-C), serum creatinine (Scr), blood urea nitrogen (BUN), serum uric acid, eGFR, and urinary protein grade (0–4 +) at baseline.

The BMI was calculated by dividing body weight in kilograms by height in meters squared and categorized into normal weight (18.5–24 kg/m^2^), overweight (24–28 kg/m^2^), and obesity (≥ 28 kg/m^2^) (Zhou 2002). Nonsmokers, current, and former smokers were categorized based on self-reported smoking status. Nonsmokers were people who had never smoked before in their lives. Current smokers were individuals who had smoked on a regular basis in the preceding six months. Former smokers were classified as those who had quit smoking for at least six months (Barry et al. 2012). Hyperlipidemia was confirmed as the usage of lipid-lowering drugs currently, the existence of any self-reported history, or meeting the requirements of any of the following circumstances: serum LDL-C ≥ 4.14 mmol/L or TG ≥ 2.26 mmol/L or HDL-C < 1.04 mmol/L or TC ≥ 6.22 mmol/L (Liu et al. 2018). Anemia was defined by the World Health Organization criteria as HGB levels below 120 g/L in women and 130 g/L in males (Kurella Tamura et al. 2016). Mean arterial pressure (MAP) was estimated by adding 1/3 of pulse pressure (systolic blood pressure [SBP]-diastolic blood pressure [DBP]) to DBP. eGFR was calculated using the Chronic Kidney Disease Epidemiology Collaboration equation (Levey et al. 2009).

### Covariates

In order to increase the credibility of the research results, we adopted the sensitivity analysis method in the multivariate logistic regression analysis. Potential covariates were determined based on clinical experience and the current literature on predictors for renal function progression in patients with T2DM and CKD (Radcliffe et al. 2017). These covariates included sex, age of DM onset, duration of DM, hyperlipidemia, the treatment of lipid-lowering drugs, smoking status, insulin treatment, HF, baseline eGFR, MAP, BMI, anemia, urinary protein, and serum ALB. Each covariate was determined at the time of the first hospitalization of each patient.

### Outcome measurements

The outcome was defined as the development of ESRD, including an eGFR of less than 15 mL/min/1.73 m^2^ or the commencement of maintenance HD, PD, or kidney transplantation. The start of observation was defined as the first hospitalization for each patient, and the endpoint was the occurrence of ESRD or the last hospitalization or clinic visit for each patient.

### Exposure assessment

The hourly measured ground concentrations of six types of air pollutants from 35 monitoring stations in Beijing since 2013 were obtained from the Beijing Municipal Ecological and Environmental Monitoring Center (http://www.bjmemc.com.cn/), including O_3_ in μg/m^3^, PM_2.5_ in μg/m^3^, PM_10_ in μg/m^3^, NO_2_ in μg/m^3^, SO_2_ in μg/m^3^, and CO in mg/m^3^. The concentration of different air pollutants was converted into the daily maximum 8-h average for O_3_ and the 24-h average for PM_2.5_, PM_10_, NO_2_, SO_2_, and CO. The locations of the air quality monitoring stations and the participant’s address (residential address) were geocoded by latitude and longitude. The ordinary Kriging method was applied to interpolate exposure concentrations onto a regular grid (400 m × 400 m) across Beijing. After transforming each participant’s address (residential address) into longitude and latitude data, using Baidu Map Open Platform (https://lbs.baidu.com), the long-term exposure of each patient was obtained. Given that the baseline exposure may not completely and accurately represent the exposure of air pollutants over the long-term follow-up, we calculated the annual mean exposure concentrations of these air pollutants between the participant’s enrollment and the last follow-up as the long-term exposure of air pollutants.

### Statistical analysis

Continuous variables conformed to the normal distribution were expressed as the mean ± standard deviation, and a *t*-test was used for comparison between the two groups, whereas continuous variables not conformed to normal distribution were presented as median (interquartile range [IQR]) and were compared using Wilcoxon test. Categorical variables were shown as percentages with the chi-squared test or Fisher’s exact test for comparison between two groups.

The Bayesian kernel machine regression (BKMR) method and the weighted quantile sum (WQS) regression were used to estimate the overall environmental pollutants (PM_2.5_, PM_10_, SO_2_, NO_2_, CO, and O_3_) on the risk of ESRD. Posterior inclusion probability (PIP) and weight were used to evaluate the importance of air pollutants. Restricted cubic spline (RCS) curves based on logistic regression were used to clarify the relationship between different air pollutants and the risk of ESRD. Univariable logistic regression analysis was performed to investigate the unadjusted association between different air pollutants and ESRD risk. Then, four multivariate logistic regression models were established as follows: model 1, adjusted for sex, age of DM onset, duration of diabetes; model 2, adjusted for variables in model 1 plus hyperlipidemia, the usage of lipid-lowering drugs, smoking status, insulin treatment, and HF; model 3, adjusted for variables in model 2 plus baseline eGFR, MAP, and BMI; model 4, adjusted for variables in model 3 plus anemia, urinary protein, and serum ALB at baseline. We further conducted subgroup analysis stratified by age (< 60 or ≥ 60 years), sex (female or male), age of DM onset (< 58 or ≥ 58 years), BMI (< 24 or ≥ 24 kg/m^2^), anemia (yes or no), hyperlipidemia (yes or no), HF (yes or no), the usage of RAAS inhibitors (yes or no), baseline eGFR (< 45 or ≥ 45 mL/min/1.73 m^2^), HbA1c (< 7 or ≥ 7%), and follow-up time (< 60 or ≥ 60 months), because these factors have been previously reported to modify the effects of air pollution (Chan et al. 2018; Chang et al. 2022) or to be associated with CKD progression in T2DM patients (Radcliffe et al. 2017; Braunwald 2019).

The correlation coefficient was used to evaluate the correlation between different air pollutants (*r* > 0.5 was considered to be statistically significant). A backward-stepwise method was used to select the optimal model. The receiver operating characteristic (ROC) curve and the area under the curve (AUC) were used to evaluate the predictive efficacy of different air pollutants to predict the risk of ESRD, and the Delong test was used to evaluate whether the difference between the two ROC curves was statistically significant. A 12000 bootstrap cohort based on the study population was used for internal validation. All analyses were performed using R (version 4.0.3).

## Results

### The primary clinical characteristics of the research population

**Fig. 2:**
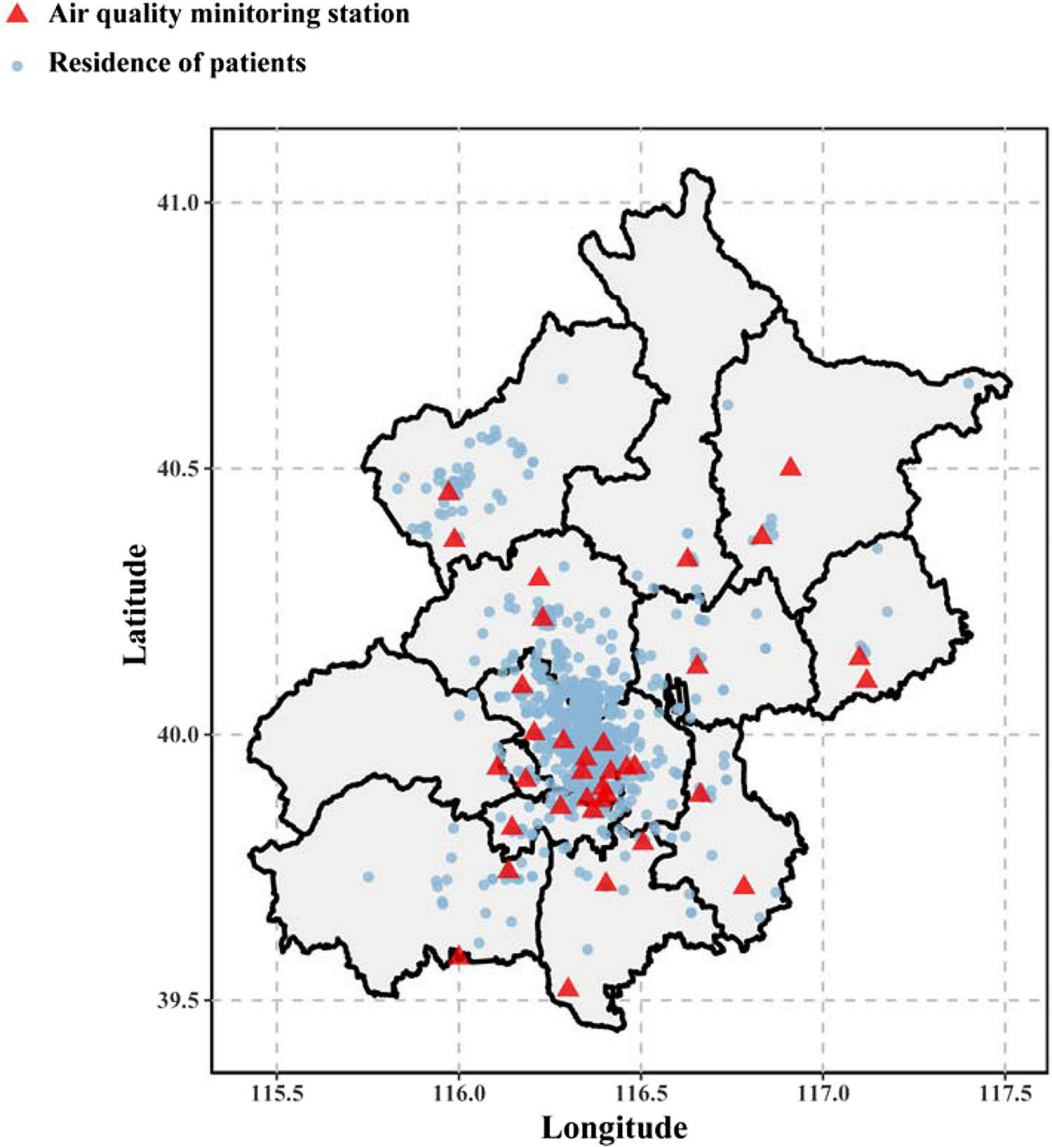
Location distribution of the study population and air quality monitoring stations in Beijing. The solid blue dot represents the location of the patient's residence, and the solid red triangle represents the location of the air quality monitoring station

All of the 1,738 patients with T2DM and CKD included in this study were permanent residents of Beijing. The location distributions of these patients and the air quality monitoring stations are depicted in Fig. 2.

The median age of the study population is 67 (59, 74) years. 1,254 (72.15%) of the patients were male and 98 (5.64%) of the patients had outcome events. Notably, compared with patients without outcomes, patients who developed ESRD had an earlier age of DM onset, a larger proportion of female patients, a longer duration of DM, a higher level of blood pressure, a higher prevalence of anemia, lower levels of serum ALB and eGFR, and higher levels of HbA1c, blood lipids, Scr, BUN, and urinary protein. The exposure differences of the five air pollutants between patients with and without outcomes were significant. Individuals who developed ESRD had higher average exposure concentrations of PM_2.5_, PM_10_, CO, NO_2_, and SO_2_ during follow-up than those without outcomes. There was no significant difference of O_3_ concentration between patients with or without outcomes. Table 1 describes the primary clinical characteristics of the research population.

**Table 1:** Baseline characteristics of the study population

Characteristics	Overall (n = 1,738)	Patients with outcomes (n = 98)	Patients without outcomes (n = 1,640)	*P*-value
**Basic clinical characteristics**				
Age (years)	67 (59, 74)	62 (55, 72)	67 (60, 74)	< 0.001
Age of DM onset (years)	66 (59, 73)	61 (53, 71)	66 (59, 73)	< 0.001
Age of DM onset category (n, %)				0.001
< 58 years	282 (16.2%)	28 (28.6%)	254 (15.5%)	
≥ 58 years	1,456 (83.8%)	70 (71.4%)	1,386 (84.5%)	
Sex (male, %)	1,254 (72.15%)	56 (57.1%)	1,198 (73%)	0.001
Duration of diabetes category (n, %)				< 0.001
< 12 months	1,157 (66.6%)	44 (44.9%)	1,113 (67.9%)	
≥ 12 months	581 (33.4%)	54 (55.1%)	527 (32.1%)	
BMI (kg/m^2^)	25.5 (23.6, 27.6)	25.3 (22.9, 27.9)	25.5 (23.7, 27.6)	0.596
BMI category (n, %)				0.286
< 24	503 (28.9%)	34 (34.7%)	469 (28.6%)	
24 – 28	857 (49.3%)	41 (41.8%)	816 (49.8%)	
≥ 28	378 (21.7%)	23 (23.5%)	355 (21.6%)	
SBP (mmHg)	135 (124, 149)	147 (125, 159)	135 (124, 148)	< 0.001
DBP (mmHg)	78 (70, 84)	81 (75, 90)	78 (70, 84)	< 0.001
MAP (mmHg)	97 (90, 104)	101 (93, 114)	96 (90, 103)	< 0.001
MAP category (n, %)				< 0.001
< 100 mmHg	1,062 (61.1%)	47 (41.8%)	1,021 (62.3%)	
≥ 100 mmHg	676 (38.9%)	57 (58.2%)	619 (37.7%)	
Smoking status (n, %)				0.604
Nonsmoker	878 (50.5%)	49 (50%)	829 (50.5%)	
Former smoker	696 (40.0%)	37 (37.8%)	659 (40.2%)	
Current smoker	164 (9.4%)	12 (12.2%)	152 (9.3%)	
**Medication history**				
Insulin (n, %)	590 (33.95%)	47 (48%)	543 (33.1%)	0.004
RAAS inhibitors (n, %)	1,027 (59.09%)	73 (74.5%)	954 (58.2%)	0.002
Lipid-lowering drugs	1,365 (78.54%)	68 (69.4%)	1,297 (79.1%)	0.032
**Main complications or comorbidities**				
Hypertension (n, %)	1,358(78.14%)	88 (89.8%)	1,270 (77.4%)	0.006
CAD (n, %)	986 (56.73%)	40 (40.8%)	946 (57.7%)	0.002
Stroke (n, %)	302 (17.38%)	20 (20.4%)	282 (17.2%)	0.498
Hyperlipidemia (n, %)	881 (50.69%)	46 (46.9%)	835 (50.9%)	0.509
HF (n, %)	198 (11.39%)	14 (14.3%)	184 (11.2%)	0.445
Anemia (n, %)	529 (30.4%)	50 (51%)	479 (29.2%)	< 0.001
**Laboratory findings**				
HGB (g/L)	134 (121, 145)	120 (106, 136)	134 (122, 146)	< 0.001
Serum ALB (g/L)	39.9 (36.9, 42.8)	36.5 (32.3, 40.2)	40.1 (37.2, 42.9)	< 0.001
ALB category (n, %)				< 0.001
< 30 g/L	91 (5.2%)	19 (19.4%)	72 (4.4%)	
≥ 30 g/L	1,647 (94.8%)	79 (80.6%)	1,568 (95.6%)	
FBG (mmol/L)	7.1 (5.9, 8.9)	7.2 (5.4, 9.5)	7.1 (5.9, 8.9)	0.97
HbA1c (%)	7.6 (6.7, 8.9)	8.0 (6.9, 10.0)	7.6 (6.7, 8.8)	0.011
HbA1c category (n, %)				0.358
< 7%	561 (32.3%)	27 (27.6%)	534 (32.6%)	
≥ 7%	1,177 (67.7%)	71 (72.4%)	1,106(67.4%)	
TC (mmol/L)	4.11 (3.42, 4.96)	4.99 (4.09, 5.57)	4.07 (3.40, 4.87)	< 0.001
TG (mmol/L)	1.62 (1.18, 2.31)	1.99 (1.48, 2.62)	1.61 (1.16, 2.30)	0.001
HDL-C (mmol/L)	0.96 (0.82, 1.12)	0.9 6(0.84, 1.20)	0.96 (0.82, 1.12)	0.630
LDL-C (mmol/L)	2.44 (1.87, 3.08)	2.76 (2.14, 3.46)	2.43 (1.86, 3.04)	0.001
LDL category (n, %)				0.003
< 2.6 mmol/L	737 (42.4%)	56 (57.1%)	681 (41.5%)	
≥ 2.6 mmol/L	1,001 (57.6%)	42 (42.9%)	959 (58.5%)	
Scr (μmol/L)	98.0 (90.0, 110.0)	112.0 (99.0, 128.0)	97.0 (90.0, 108.3)	< 0.001
BUN (mmol/L)	6.8 (5.6, 8.2)	8.5 (6.6, 10.4)	6.7 (5.5, 8.1)	< 0.001
Serum uric acid (μmol/L)	381 (324, 445)	387 (331, 467)	381 (323, 444)	0.255
eGFR (mL/min/1.73 m^2^)	52.1 (45.0, 57.5)	45.0 (37.7,53.6)	52.3 (45.4, 57.6)	< 0.001
eGFR category (n, %)				< 0.001
Stage 1 (≥ 90)	56 (3.2%)	2 (2%)	54 (3.3%)	
Stage 2 (60 – 89)	133 (7.7%)	11 (11.2%)	122 (7.4%)	
Stage 3a (45 – 59)	1,113 (64%)	36 (36.7%)	1,077 (65.7%)	
Stage 3b (30 – 44)	436 (25.1%)	49 (50%)	387 (23.6%)	
Urinary protein grade (n, %)				< 0.001
0 – 1 +	1,241 (71.4%)	20 (20.4%)	1,221 (74.5%)	
2 + – 4 +	497 (28.6%)	78 (79.6%)	419 (25.5%)	
PM_2.5_ (µg/m^3^)	49.50 (38.67, 62.36)	55.55 (43.46, 66.02)	49.22 (38.32, 62.01)	0.002
PM_10_ (µg/m^3^)	81.40 (69.17, 94.58)	85.14 (74.34, 97.75)	81.12 (68.80, 94.09)	0.016
CO (mg/m^3^)	0.81 (0.65, 0.99)	0.88 (0.72, 1.06)	0.80 (0.64, 0.98)	0.005
NO_2_ (µg/m^3^)	40.93 (33.25, 47.68)	42.27 (36.02, 49.78)	40.81 (33.15, 47.58)	0.049
SO_2_ (µg/m^3^)	5.96 (3.74, 8.93)	7.36 (5.05, 10.20)	5.91 (3.70,8.84)	0.001
O_3_ (µg/m^3^)	92.74 (90.26, 94.96)	93.01 (91.57, 94.79)	92.71 (90.18, 94.99)	0.394

Abbreviations: DM: diabetes mellitus; BMI: body mass index; RAAS: renin–angiotensin–aldosterone system; CAD: coronary artery disease; HF: heart failure; SBP: systolic blood pressure; DBP: diastolic blood pressure; MAP: mean arterial pressure; HGB: hemoglobin; ALB: albumin; FBG: fasting blood glucose; HbA1c: glycated hemoglobin; TC: total cholesterol; TG: triglyceride; HDL-C: high-density lipoprotein cholesterol; LDL-C: low-density lipoprotein cholesterol; Scr: serum creatinine; BUN: blood urea nitrogen; eGFR: estimated glomerular filtration rate; PM_2.5_: particulate matter with an aerodynamic diameter less than 2.5 μm; PM_10_: particulate matter with an aerodynamic diameter less than 10 μm; CO: carbon monoxide; NO_2_: nitrogen dioxide; SO_2_: sulfur dioxide. O_3_: ozone

### Association between different air pollutants and the risk of ESRD

**Fig. 3:**
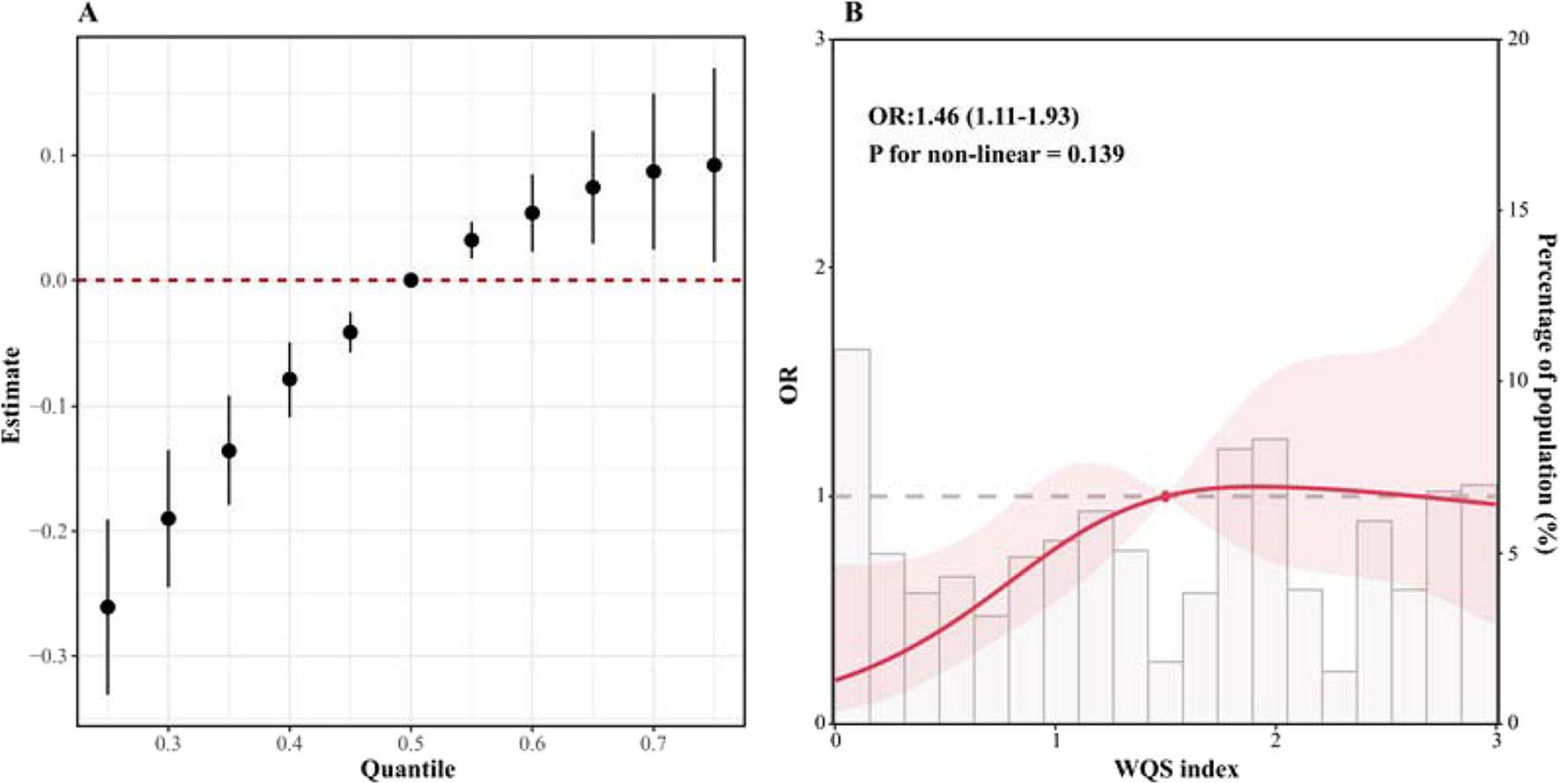
Overall effects of environmental pollutants including PM_2.5_, PM_10_, SO_2_, CO, NO_2_, and O_3_. A: The BKMR method showed the estimated change in risk of ESRD when six pollutants were set at particular percentiles (ranging from 25 to 75th) compared to when all pollutants were all at their 50th percentile; B: RCS curve of WQS index, which was calculated by WQS regression, indicating the overall effort of all the six environmental pollutants

The results of the BKMR method showed that the estimated risk of ESRD increased with a simultaneous increase of the six air pollutants, from 25th percentile to 75th percentile after adjusting for sex, age of DM onset, duration of diabetes, hyperlipidemia, using of lipid-lowering drugs, smoking status, insulin treatment, HF, baseline eGFR, MAP, BMI, anemia, urinary protein, and serum ALB, indicating a positive joint effect of pollutant mixtures (Fig. 3A). The WQS regression also showed that the WQS index of overall air pollutants was positively associated with ESRD risk (odds ratio [OR] = 1.46, 95%confidence interval [CI] 1.11–1.93). The RCS curve showed a linear relationship between the WQS index and the risk of ESRD (Fig. 3B).

Fig. S1 demonstrated the univariate concentration–response functions and 95% CIs for each pollutant with the other pollutants fixed at the median values. We observed a significantly increasing concentration–response relationship for PM_2.5_, PM_10_, SO_2_, and CO. A significantly decreasing concentration–response relationship was observed for NO_2_, while O_3_ has no significantly relationship with the risk of ESRD. The interactive analysis of BKMR method showed that other environmental pollutants (PM_10_, SO_2_, CO, and O_3_) have different influence towards ESRD when PM_2.5_ and NO_2_ were at different level, indicating there was a interactive effect (Fig. S2).

**Fig. 4:**
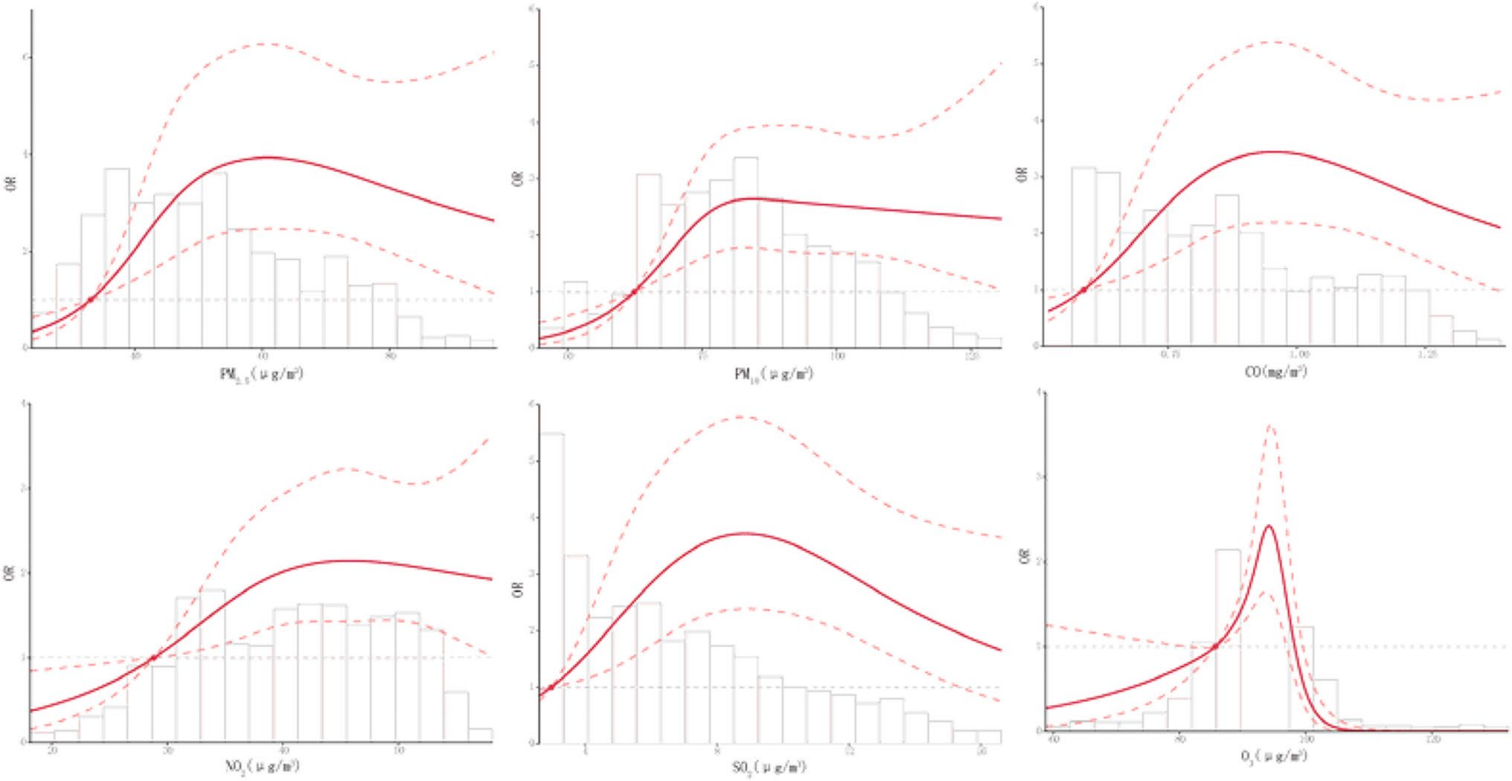
Association between the risk of ESRD and different air pollutants. The solid red line represented OR of the air pollutant across the whole range after fully adjusting for covariates, including sex, age of DM onset, duration of diabetes, hyperlipidemia, using of lipid-lowering drugs, smoking status, insulin treatment, HF, baseline eGFR, MAP, BMI, anemia, urinary protein, and serum ALB. The red dot line represents the 95% CI of OR. The frequency distribution of the air pollutant concentration is shown by histograms

In the dose–response analysis, we used the RCS curves to describe the relationship between six types of air pollutants and ESRD risk. After fully adjusting for covariates, including sex, age of DM onset, duration of diabetes, hyperlipidemia, using of lipid-lowering drugs, smoking, insulin treatment, HF, baseline eGFR, MAP, BMI, anemia, urinary protein, and serum ALB, a positive relationship was found between five types of air pollutants (PM_2.5_, PM_10_, CO, NO_2_, and SO_2_) and the risk of ESRD, while O_3_ showed no significant association (Fig. 4).

In the univariate logistic regression analysis, the most substantial crude effect was found in the association between an increase of 1 mg/m^3^ in CO during follow-up and the risk of developing ESRD (OR = 3.14, 95% CI 1.03–1.36, *P* = 0.013), followed by an increase of 10 μg/m^3^ in PM_2.5_ (OR = 1.20, 95% CI 1.06–1.36, *P* = 0.004). Other air pollutants (PM_10_ and SO_2_) were also found to have a significantly positive association with ESRD risk, except for O_3_ and NO_2._ Furthermore, four multivariate adjustment models were established in this study. After fully adjusting for potential confounders, the positive association between PM_2.5_, PM_10_, CO, and SO_2_ and the risk of ESRD remained robust. Details of the univariate and multivariate logistic regression results are presented in Table 2.

**Table 2:** OR (95% CI) of ESRD risk associated with six air pollutants (PM_2.5_, PM_10_, CO, NO_2_, SO_2_, and O_3_)

	PM_2.5_ (per 10 μg/m^3^)		PM_10_ (per 10 μg/m^3^)		CO (per 1 mg/m^3^)		NO_2_ (per 10 μg/m^3^)		SO_2_ (per 1 μg/m^3^)		O_3_ (per 1 μg/m^3^)	
	OR (95% CI)	*P*-value	OR (95% CI)	*P*-value	OR (95% CI)	*P*-value	OR (95% CI)	*P*-value	OR (95% CI)	*P*-value	OR (95% CI)	*P*-value
Unadjusted model	1.20 (1.06–1.36)	0.004	1.16 (1.03–1.30)	0.013	3.14 (1.27–7.78)	0.013	1.25 (0.99–1.59)	0.061	1.08 (1.02–1.14)	0.006	1.00 (0.76, 1.31)	0.978
Model 1	1.19 (1.05–1.35)	0.007	1.15 (1.02–1.29)	0.021	3.05 (1.21–7.67)	0.018	1.25 (0.98–1.59)	0.066	1.07 (1.01–1.13)	0.013	0.97 (0.73, 1.27)	0.799
Model 2	1.17 (1.03–1.33)	0.015	1.13 (1.01–1.27)	0.039	2.77 (1.09–7.05)	0.033	1.21 (0.95–1.54)	0.120	1.07 (1.01–1.13)	0.024	0.95 (0.72, 1.25)	0.716
Model 3	1.18 (1.03–1.34)	0.014	1.14 (1.01–1.29)	0.030	2.75 (1.06–7.11)	0.037	1.22 (0.96–1.57)	0.109	1.07 (1.01–1.14)	0.022	0.93 (0.71, 1.23)	0.614
Model 4	1.19 (1.04–1.36)	0.013	1.15 (1.02–1.31)	0.022	2.80 (1.05–7.47)	0.040	1.26 (0.98–1.61)	0.073	1.07 (1.00–1.13)	0.040	0.86 (0.65, 1.13)	0.278

Model 1 adjusted for sex, age of DM onset, and duration of diabetes; Model 2 adjusted for variables in model 1 plus hyperlipidemia, using of lipid-lowering drugs, smoking status, insulin treatment, and HF; Model 3 adjusted for variables in model 2 plus baseline eGFR, MAP, and BMI; Model 4 adjusted for variables in Model 3 plus anemia, urinary protein, and serum ALB at baseline. Abbreviations: OR: odds ratio; CI: confidence interval; ESRD: end-stage renal disease; PM_2.5_: particulate matter with an aerodynamic diameter less than 2.5 μm; PM_10_: particulate matter with an aerodynamic diameter less than 10 μm; CO: carbon monoxide; NO_2_: nitrogen dioxide; SO_2_: sulfur dioxide; O_3_: ozone. DM: diabetes mellitus; HF: heart failure; eGFR: estimated glomerular filtration rate; MAP: mean arterial pressure; BMI: body mass index; ALB: albumin

To clarify which environmental pollutant has the most significant impact on ESRD risk, we calculated the posterior inclusion probability (PIP) using BKMR method and weights using WQS regression. In the BKMR model, PM_2.5_ has the highest PIP value (0.688). In the WQS regression, PM_2.5_ also has the highest weight (0.428), indicating that PM_2.5_ has a stronger correlation with ESRD risk among the six types of air pollutants (Fig. S3).

### Subgroup analysis

**Fig. 5:**
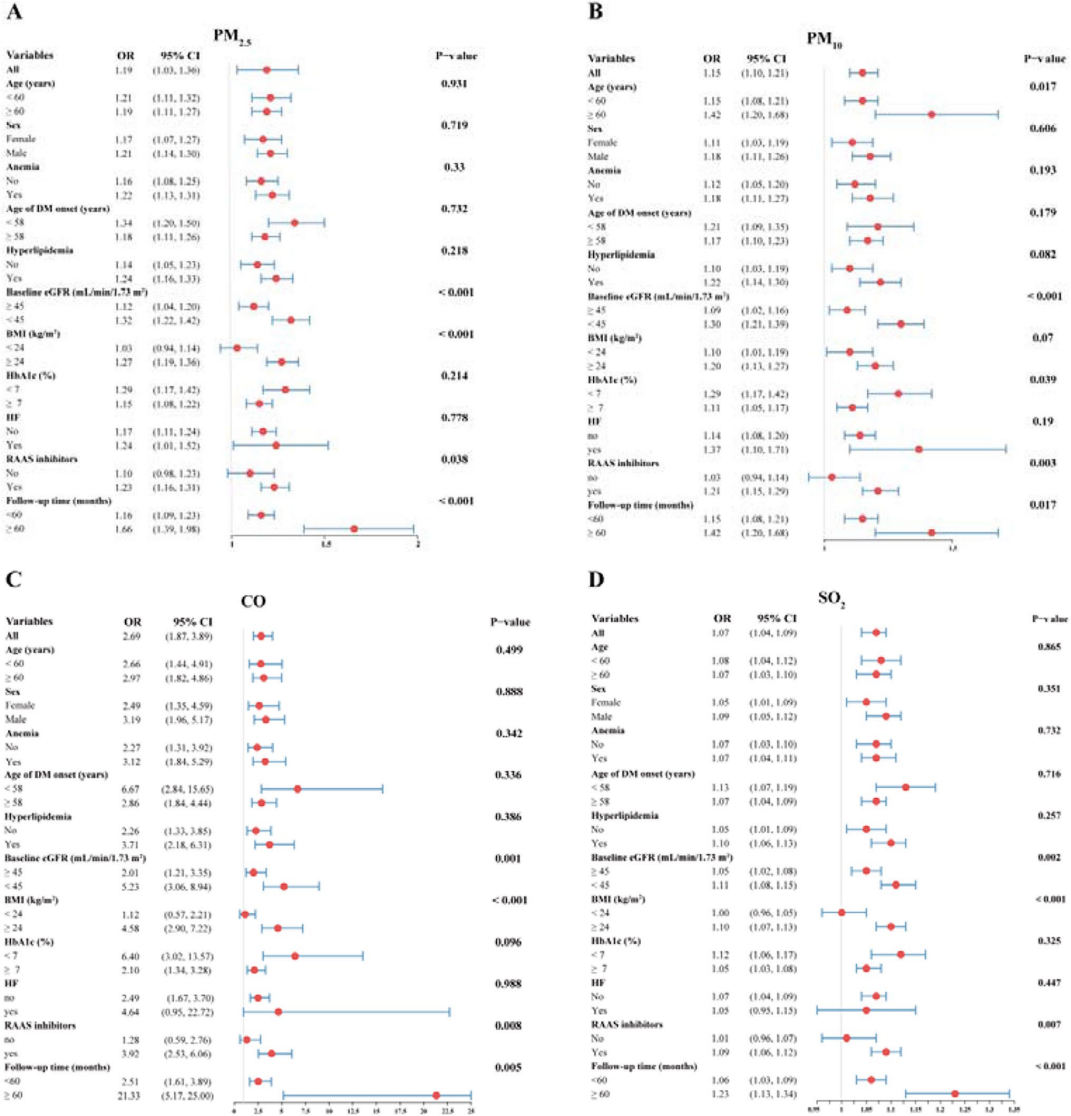
The associations between long-term exposure to PM_2.5_, PM_10,_ CO, and SO_2_ and ESRD risk in patients with different subgroups

Figure 5 presented the associations between PM_2.5_, PM_10_, CO, and SO_2_ exposure and the risk of ESRD based on age, sex, age of DM onset, anemia, hyperlipidemia, HF, the usage of RAAS inhibitors, baseline eGFR, BMI, HbA1c, and follow-up time. A 10 μg/m^3^ increment in PM_2.5_ during the follow-up period was associated with higher increased odds of developing ESRD in patients with a lower baseline eGFR (OR 1.32 *vs*. 1.12, *P* for interaction < 0.001), a higher BMI (OR 1.27 *vs*. 1.03, *P* for interaction < 0.001), the usage of RAAS inhibitors (OR 1.23 *vs*. 1.10, *P* for interaction = 0.038), and a longer follow-up period (OR 1.66 *vs*. 1.16, *P* for interaction < 0.001), similar results were also found in CO and SO_2_. Apart from baseline eGFR, usage of RAAS inhibitors, and follow-up time, a 10 μg/m^3^ increment in PM_10_ during the follow-up period was also found to be associated with a higher increased odds of incident ESRD in patients with an age ≥ 60 years (OR 1.42 *vs.* 1.15, *P* for interaction = 0.017) and HbA1c < 7% (OR 1.29 *vs.* 1.11, *P* for interaction = 0.039).

**Fig. 6:**
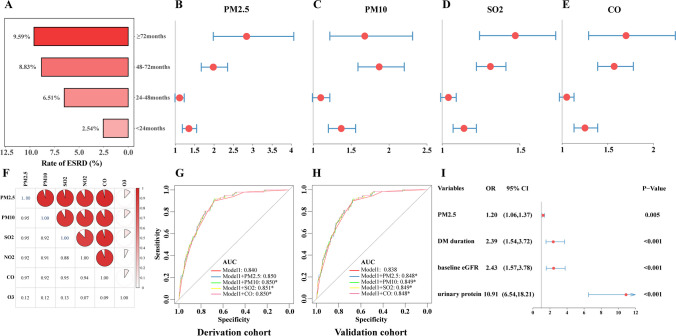
Subgroup analysis of follow-up time and evaluation of prognostic efficacy of different air pollutants. A-E. Subgroup analysis of follow-up time in the association between PM_2.5_, PM_10_, SO_2_, or CO and the risk of developing ESRD; F. Correlation analysis between different air pollutants; G-H. The prognostic performance of different air pollutants for ESRD in the derivation and internal validation cohort. I. The optimal model established by a backward stepwise method based on PM_2.5_, PM_10_, SO_2_, CO, and variables in model 4

As shown in Fig. 5, It is noteworthy that whether the follow-up time was shorter (< 60 months) or longer (≥ 60 months), exposure to PM_2.5_, PM_10_, CO, and SO_2_ were all positively associated with an increased risk of ESRD. Further analysis found that, regardless of the duration of follow-up, long-term exposure to PM_2.5_, PM_10_, SO_2_, and CO could increase the risk of ESRD (Fig. 6B-E), and the incidence of ESRD gradually increased with the prolongation of follow-up time (Fig. 6A).

### Evaluation of the prognostic performance of different air pollutants for ESRD

Since there were strong correlations (*r* > 0.5) between each of the five air pollutants except O_3_ (Fig. 6F), we first evaluated the diagnostic efficacy of each air pollutant separately. Air pollution indicators could significantly improve the predictive performance of the basic clinical model (model 1, including sex, age of DM onset, duration of DM, hyperlipidemia, the treatment of lipid-lowering drugs, smoking status, insulin treatment, HF, baseline eGFR, MAP, BMI, anemia, urinary protein, and serum ALB), except for PM_2.5_ (0.850 *vs.* 0.840, *P* > 0.05, Fig. 6G). In the bootstrap cohort, air pollution indicators, including PM_2.5_, can significantly improve the predictive performance of the basic model (Fig. 6H). In order to identify the air pollution with the strongest prognostic efficacy for ESRD and establish the optimal model, we used the backward stepwise method for variable screening, including PM_2.5_, PM_10_, SO_2_, CO, and variables in model 1. Finally, four indicators were included in the optimal model, with PM_2.5_ being the most effective environmental indicator among all the air pollutants (Fig. 6I).

## Discussion

In the current study, we investigated the association between long-term exposure to six types of ambient air pollutants (PM_2.5_, PM_10_, CO, NO_2_, SO_2_, and O_3_) and the risk of ESRD in Chinese patients with T2DM and CKD. We confirmed the significant long-term risks of PM_2.5_, PM_10_, CO, and SO_2_ for progression to ESRD. These positive associations remained robust in fully adjusted models, suggesting these associations were independent. Different effects were shown by different air pollutants when stratified by age, baseline eGFR, BMI, the usage of RAAS inhibitors, HbA1c, and follow-up time. Furthermore, on the basis of the clinical model incorporating traditional predictors (model 1), adding the above four types of air pollutants (PM_2.5_, PM_10_, CO, or SO_2_) can significantly improve the predictive efficiency of the model for predicting ESRD. Using BKMR method, WQS regression, and backward stepwise logistic regression, we found that PM_2.5_ was the most important environmental predictor for ESRD, indicating the importance of incorporating PM_2.5_ exposure into the regular clinical care of patients with T2DM and CKD.

A recent prospective cohort study including 6,628 adult patients with CKD investigated the association between long-term exposure to PM_2.5_ and the risk of ESRD and found that the adjusted hazard ratio (95% CI) for progression to ESRD was 1.19 (1.08–1.31) per 7.8 μg/m^3^ increment in PM_2.5_, which was similar to our results (Lin et al. 2020b). However, this study did not investigate other air pollutants and included the general population with CKD. In our study, apart from PM_2.5_, we also explored the association between five other types of air pollutants (PM_10_, CO, NO_2_, SO_2_, and O_3_) and the risk of ESRD and further confirmed the significantly negative impact of long-term exposure to PM_10_, CO, and SO_2_ on renal function. It is noteworthy that our study focused on a specific group of patients with T2DM and CKD, which was reported to have a significantly higher incidence rate of chronic RRT than people without diabetes (Claessen et al. 2021) and also have a higher mortality rate (Alicic et al. 2017). Additionally, at present, there is limited epidemiological evidence of the association between air pollution and ESRD risk in patients with T2DM and CKD. Therefore, our study provided an evidentiary base to incorporate PM_2.5_, PM_10_, CO, and SO_2_ exposure into the regular clinical care of patients with T2DM and CKD.

Given that the baseline exposure to air pollution may not completely and accurately represent the exposure over the long-term follow-up, we used the annual mean exposure concentrations of different air pollutants between the patient’s enrollment and the last follow-up, which was similar to several previous studies using ESRD as the endpoint (Bowe et al. 2017, 2018; Lin et al. 2020a). Furthermore, we discussed the impact of the duration of follow-up on the association between air pollution and the risk of ESRD. We found that the negative impact of air pollution on renal function remained significant regardless of the duration of follow-up, and these effects tended to be stronger in those with a relatively long follow-up period, indicating that reducing exposure time is of great significance for delaying ESRD progression in patients with T2DM and CKD.

Moreover, our study investigated the additional predictive value of PM_2.5_, PM_10_, CO, and SO_2_ outperforming the conventional clinical model (model 1) both in the derivation and the internal validation cohort. After using the BKMR method, WQS regression, and the backward stepwise logistic regression to select the variables, PM_2.5_ was found to be the most important environmental pollutant. Previous studies suggested that ambient PM_2.5_ could be a novel environmental risk factor for incident CKD or ESRD (Bowe et al. 2018; Lin et al. 2020a, b; Blum et al. 2020; Yang et al. 2017; Chang et al. 2022; O'Neill et al. 2008; Mehta et al. 2016; Weaver et al. 2019). However, limited data exist on the association between long-term exposure to PM_2.5_ and ESRD risk in countries or regions with high levels of PM_2.5_, especially in the Chinese mainland, where high levels of PM_2.5_ remain a very severe environmental challenge (Huang et al. 2018). Our research findings may further promote public health efforts to provide greater protection for patients with T2DM and CKD in reducing ESRD risks associated with long-term exposure to PM_2.5_ and provide evidence to implement more reinforced air quality control of ambient PM_2.5_.

Previous studies indicated that ambient air pollutants might cause renal vascular impairment, mesangial expansion, intraglomerular hypertension, advanced glomerulosclerosis, tubular atrophy, and renal fibrosis, which may contribute to the progression of kidney diseases (Yan et al. 2014; Tavera Busso et al. 2018; Al Suleimani et al. 2017). The mechanisms underlying air pollutants to renal function decline are not entirely clear and may be involved in many aspects. For example, PM_2.5_ can enter the lungs and further pass into the bloodstream, and then penetrate blood-organ barriers and thereby impact distant organs like the kidneys (Chen et al. 2022). Experimental evidence suggested that PM_2.5_ could cause disturbances of renal hemodynamics, exacerbate renal vascular damage, aggravate oxidative stress and inflammation, promote DNA damage, and thereby promote the development of acute kidney injury or CKD (Al Suleimani et al. 2017; Nemmar et al. 2010, 2016). Future studies are still needed to elucidate the mechanisms by which different air pollutants affect renal function.

In this study, we included patients with T2DM and CKD. Notably, air pollution is also a major risk factor for T2DM, a leading driver of CKD. According to an analysis of data from the Global Burden of Disease Study 2019, about 20% of the global burden of T2DM can be attributed to ambient PM_2.5_ exposure, which contributes to 13.4% (9.49–17.5) of deaths and 13.6% (9.73–17.9) of disability-adjusted life-years due to T2DM (GBD 2019 Diabetes and Air Pollution Collaborators 2022). A recent study included a cohort of 2,444,157 veterans from the United States and investigated whether DM mediates the association between PM_2.5_ and CKD (Bowe et al. 2020). They found that DM mediates a certain proportion of the positive association between PM_2.5_ and the risk of various kidney outcomes, especially ESRD, suggesting that more precise estimates of the burden of DM and the burden of kidney disease attributable to ambient PM_2.5_ will be considered in future studies.

Notablely, apart from the covariates that we have already adjusted in this study, there are some potential confounding factors, such as genetic factors, diet, and indoor air pollution, which were also previously reported to be associated with renal function decline. Genetic polymorphisms and epigenetic variations were found to determine the individual susceptibility to renal function progression (Tampe and Zeisberg 2014). In addition, genetic risk was also reported to modify the association between air pollutants exposure and CKD (Wang et al. 2022). Healthy diets, such as coffee, dairy, and plant-based foods may lower the risk of CKD, while unhealthy diets, such as sugar-sweetened beverages and red meat, may accelerate renal function decline (van Westing et al. 2020). Moreover, there are several studies exploring the association between indoor air pollution and renal function progression, which have yielded inconsistent results (Singh et al. 2016; Xue et al. 2022; Kanagasabai et al. 2022). In our study, we could not further analyze these confounding factors due to data inavailability. Future studies are needed to further explore the impact of these confounding factors on renal function in patients with T2DM and CKD.

Based on a retrospective cohort in Beijing, China, we first provided an evidentiary base for the positive association between long-term exposure to different air pollutants and the risk of ESRD in Chinese patients with T2DM and CKD. Moreover, we evaluated the additional predictive value for ESRD outperforming the conventional clinical model. However, our study also has several limitations. First, this is a single-center study with a relatively small sample size, which may affect the generalizability of our results to a broader population with T2DM and CKD. Second, this is a retrospective cohort study, which may suffer from bias in report and selection. Research with a prospective design is warranted in future studies. Third, we applied the ordinary Kriging method to estimate exposure from air pollutant concentration of the air quality monitoring station nearest the participant’s residential address. We could not fully consider the daily activity trajectory of the subjects due to the limitation of data. Fourth, we were unable to further distinguish patients with diabetic nephropathy and nondiabetic kidney disease (NDKD) due to the lack of renal biopsy data. Future studies are needed to further compare the different effects of air pollution on the progression of diabetic nephropathy and NDKD.

## Conclusions

Our study evaluated the long-term effects of ambient PM_2.5,_ PM_10_, NO_2_, CO, SO_2_, and O_3_ on ESRD risk in Chinese patients with T2DM and CKD and revealed a significantly positive association between PM_2.5,_ PM_10_, CO, and SO_2_ and risk of ESRD. These associations remained robust after multiple adjustments. Besides, these air pollution indicators showed additional predictive value outperforming the conventional clinical model, and PM_2.5_ was found to be the most important environmental pollutant. Therefore, our findings support the independent association between long-term exposure to different air pollutants and the risk of ESRD and provide an evidentiary base to incorporate air pollutant exposure, especially PM_2.5_, into the regular clinical care of patients with T2DM and CKD.

### Supplementary Information

Below is the link to the electronic supplementary material.

Supplementary file1 (DOC 913 KB)

## Data Availability

The data of this study are available on request from the corresponding author.

## Abbreviations

ESRD: End-stage renal disease
T2DM: Type 2 diabetes mellitus
CKD: Chronic kidney disease
OR: Odds ratio
CI: Confidence interval
DM: Diabetes mellitus
RRT: Renal replacement therapy
HD: Hemodialysis
PD: Peritoneal dialysis
PM: Particulate matter
PM_2.5_: Particulate matter with an aerodynamic diameter less than 2.5 μm
PM_10_: Particulate matter with an aerodynamic diameter less than 10 μm
NO_2_: Nitrogen dioxide
CO: Carbon monoxide
SO_2_: Sulfur dioxide
T1DM: Type 1 diabetes mellitus
GDM: Gestational diabetes mellitus
eGFR: Estimated glomerular filtration rate
BMI: Body mass index
RAAS: Renin-angiotensin-aldosterone system
CAD: Coronary artery disease
HF: Heart failure
HGB: Hemoglobin
ALB: Albumin
FBG: Fasting blood glucose
HbA1c: Glycated hemoglobin
TC: Total cholesterol
TG: Triglyceride
HDL-C: High-density lipoprotein cholesterol
LDL-C: Low-density lipoprotein cholesterol
Scr: Serum creatinine
BUN: Blood urea nitrogen
MAP: Mean arterial pressure
SBP: Systolic blood pressure
DBP: Diastolic blood pressure
IQR: Interquartile range
RCS: Restricted cubic spline
ROC: Receiver operating characteristic
AUC: Area under the curve
NDKD: Nondiabetic kidney disease
BKMR: Bayesian kernel machine regression
WQS: Weighted quantile sum
PIP: Posterior inclusion probability
