# Supplementary material for: Long-term exposure to ambient air pollutants and increased risk of end-stage renal disease in patients with type 2 diabetes mellitus and chronic kidney disease: a retrospective cohort study in Beijing, China
Source: Environ Sci Pollut Res Int. 2023 Dec 20;31(4):5429–43. doi: 10.1007/s11356-023-31346-2 (PMC10799089; doi:10.1007/s11356-023-31346-2)
Supplement: Supplementary file 1 — Supplementary file1 (DOC 913 KB) [file 11356_2023_31346_MOESM1_ESM.doc]

Supplementary Material


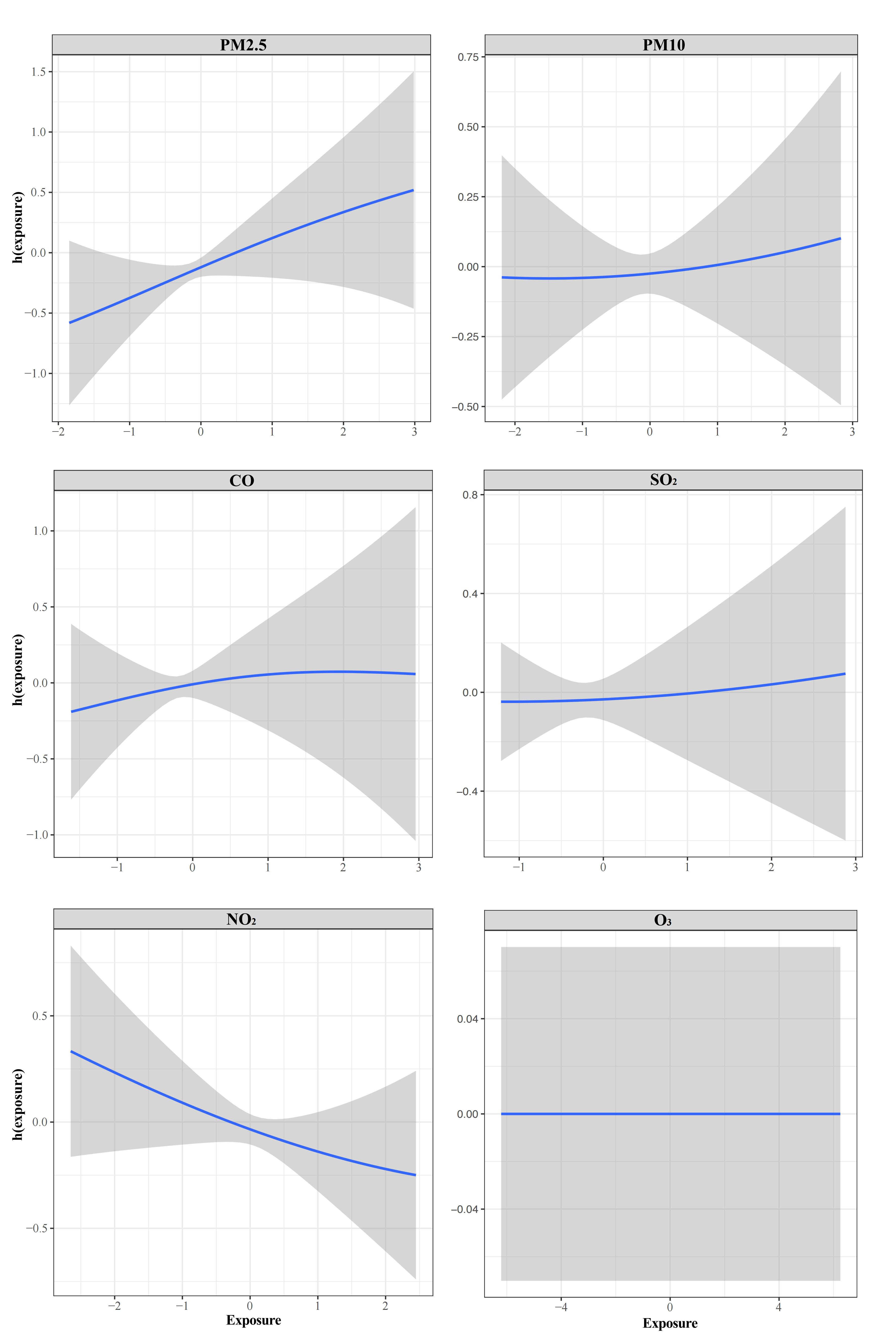


**Fig. S1** The univariate concentration-response functions with 95% confidence bands (shaded areas) for each pollutant (PM_2.5_, PM_10_, CO, SO_2_, NO_2_, and O_3_) with the other pollutants fixed at the median.


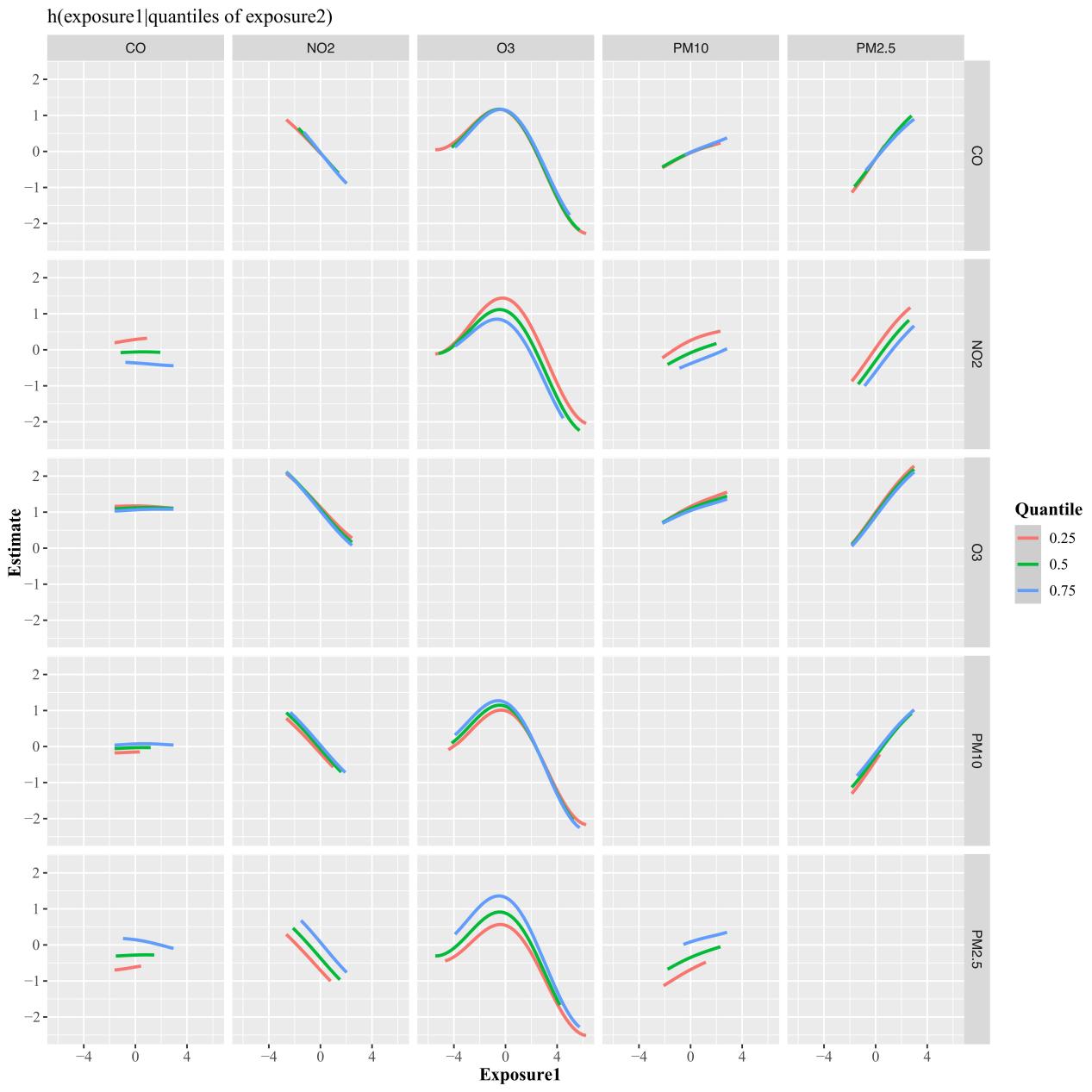


**Fig. S2** Bivariate exposure response functions for each pollutant presented on x-axis when pollutant on y-axis was fixed at 25% (red line), 50% (green line), and 75% (blue line) percentile respectively, and other pollutants were fixed at their median.


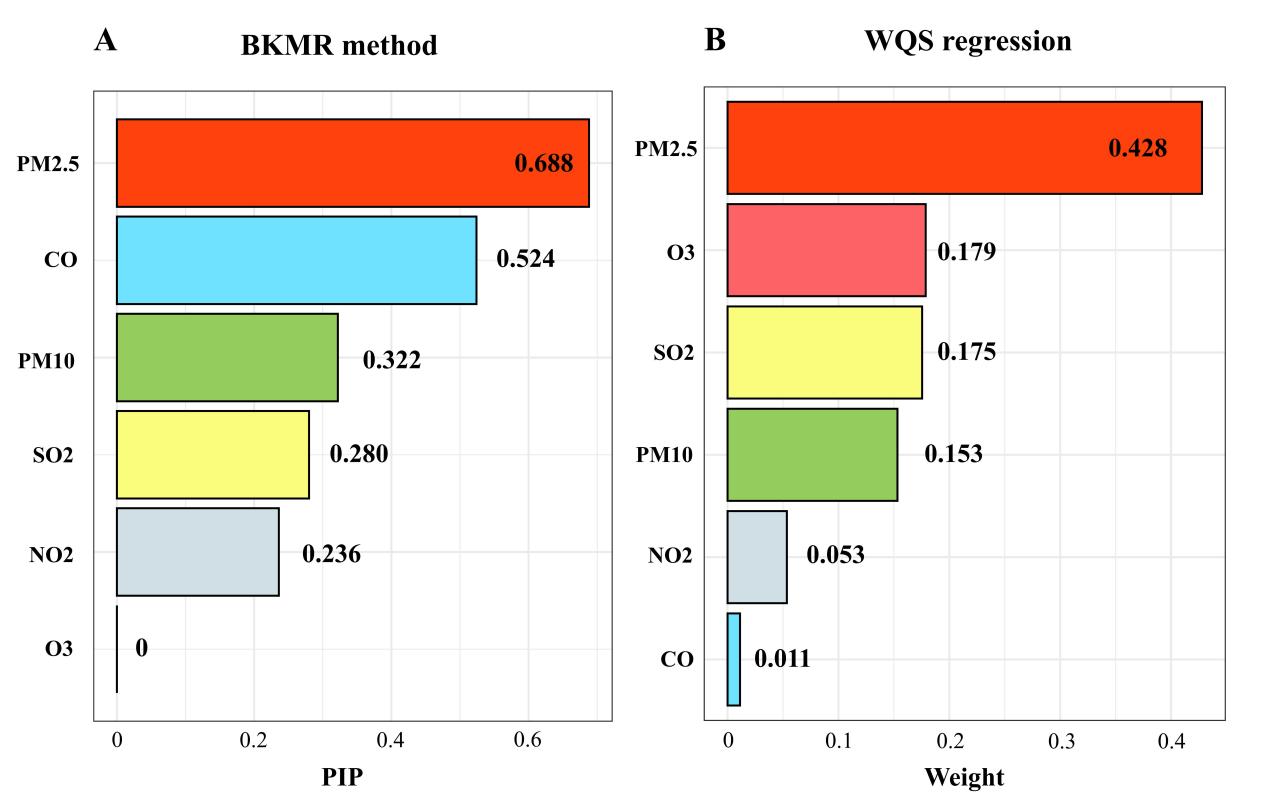


**Fig. S3** PIPs and weights of environmental pollutants by BKMR method (A) and WQS regression (B).
